# Combined Action Observation and Motor Imagery Elicits Superior Frontoparietal Activation in Elite Ski Jumpers: An fNIRS Study

**DOI:** 10.3390/brainsci16060629

**Published:** 2026-06-11

**Authors:** Qing Yan, Keying Zhang, Yuyan Wang, Haibin Zhou, Ling Jiang, Chunmei Cao, Laikang Yu, Dong Zhang

**Affiliations:** 1 Institute of Artificial Intelligence in Sports, Capital University of Physical Education and Sports, Beijing 100191, China; 13181579677@163.com (Q.Y.); 18836225802@163.com (Y.W.); 13573274238@163.com (H.Z.); 2 Department of Physical Education, Southeast University, Nanjing 211189, China; bsuzky0812@163.com (K.Z.); l1ng_jiang@163.com (L.J.); 3 Division of Sports Science and Physical Education, Tsinghua University, Beijing 100084, China; caocm@tsinghua.edu.cn; 4 Department of Strength and Conditioning Assessment and Monitoring, Beijing Sport University, Beijing 100084, China; yulaikang@126.com

**Keywords:** action observation, motor imagery, fNIRS, ski jumping, frontoparietal network, neural efficiency, cognitive training

## Abstract

**Highlights:**

**What are the main findings?**

- Concurrent action observation and motor imagery (AO + MI) elicited significant cortical activation in elite ski jumpers, primarily in the precentral gyrus, postcentral gyrus, and middle frontal gyrus, whereas AO or MI alone did not produce significant activation.
- Condition-wise comparisons showed that AO + MIgenerally evoked stronger hemodynamic responses than AO or MI across several frontoparietal channels, including the inferior parietal lobule and supramarginal gyrus, suggesting that simultaneous AO + MI may engage broader neural networks during complex whole-body movements.

**What are the implications of the main findings?**

- The observed activation in precentral, postcentral, and middle frontal regions may reflect the neural mechanisms underlying critical aspects of ski jumping, including whole-body coordination, motor planning, and visuomotor integration, providing insight into the cortical substrates supporting execution of high-level, complex movements in elite athletes.
- The stronger engagement under AO + MIindicates that combining action observation and motor imagery could serve as a useful approach for probing these neural processes, offering a framework for future studies on cognitive–motor strategies and training optimization in ski jumping and similar high-performance sports.

**Abstract:**

**Background**: Action observation (AO) and motor imagery (MI) are widely used cognitive training strategies. Recent evidence suggests that their combination may enhance motor simulation through synergistic neural mechanisms. However, the effects of this approach on complex whole-body movements in elite athletes remain unclear. **Methods**: Twenty-seven elite ski jumpers performed AO, MI, and concurrent AO + MI tasks, while cortical hemodynamic responses were recorded using functional near-infrared spectroscopy (fNIRS). Channel-level changes in oxygenated hemoglobin (ΔHbO) were analyzed using one-sample and paired-sample *t*-tests, with false discovery rate (FDR) correction applied for multiple comparisons. Additionally, mixed-design ANOVAs were conducted to examine the potential modulation of athlete level (master-level vs. first-class). **Results**: Significant activation was observed only in the AO + MIcondition after FDR correction, primarily in channels corresponding to the precentral gyrus, postcentral gyrus, and middle frontal gyrus. No channels in the AO and MI conditions survived FDR correction. Between-condition comparisons revealed significant differences in several channels located in frontoparietal regions, including the inferior parietal lobule, supramarginal gyrus, and middle frontal gyrus, with AO + MIgenerally showing stronger responses. No significant effects related to athlete level were found. **Conclusions**: These findings indicate that concurrent AO + MI is more effective than AO or MI alone in eliciting cortical activation in elite ski jumpers. This may reflect enhanced engagement of frontoparietal networks involved in action representation and visuomotor integration. These results may be compatible with a neural efficiency interpretation in highly trained athletes, although further studies with behavioral outcomes and broader skill-level comparisons are needed. AO + MI may represent a promising strategy for off-snow cognitive training in high-risk sports.

## 1. Introduction

Ski jumping is a high-risk sport that requires precise whole-body coordination within seconds. The take-off and early flight phases are critical determinants of performance [1,2]. However, frequent on-snow practice is often limited by facility availability, weather conditions, and safety concerns [3,4,5]. This underscores the need for repeatable, safe, and controllable off-snow training methods to reinforce motor patterns and refine technique.

Action observation (AO) and motor imagery (MI) are two widely used cognitive training approaches. MI refers to the mental simulation of a movement without overt motor output [6], and has been shown to engage sensorimotor networks, including the premotor and primary motor cortices [7]. AO involves watching movements, typically through live demonstrations or videos. During AO, a mirror neuron system, encompassing the inferior frontal gyrus and superior temporal sulcus, is engaged via visuomotor neural circuits [8,9]. According to mental simulation theory, both AO and MI recruit neural networks that partially overlap with those underlying actual motor execution. A meta-analysis by Hardwick et al. confirmed that both tasks consistently activate the bilateral dorsal and ventral premotor cortex, supplementary motor area, and parietal regions, supporting their role in motor learning and performance enhancement [7].

Despite their theoretical foundation, the effectiveness of AO or MI alone has shown considerable variability, with outcomes depending on task complexity, skill level, and training duration [8]. In response to this variability, recent studies have proposed combining both approaches. The dual action simulation hypothesis (DASH) posits that when visual observation and kinesthetic imagery are directed toward the same action, two parallel streams of information converge and potentiate each other, resulting in synergistic neural activation [9,10]. Emerging evidence indicates that simultaneous AO + MI enhances corticospinal excitability more than either method alone, as assessed via transcranial magnetic stimulation [10], and may improve behavioral outcomes in motor performance [11]. However, the effects ofAO + MI should not be assumed to be universally superior to those of AO or MI alone, as they may depend on task characteristics, participant expertise, imagery ability, and the correspondence between observed and imagined actions [10,12,13]. More specifically, AO + MI-related benefits may be stronger when the observed and imagined actions are temporally and spatially congruent, when participants can maintain vivid kinesthetic imagery during observation, and when the task requires the integration of external visual information with internally generated motor representations [10,12,13].

Ski jumping is particularly suitable for examining this issue because it requires rapid whole-body coordination, postural control, timing precision, and sensorimotor prediction under high-risk conditions. Building on these sport-specific demands, ski jumping provides a useful model for examining how AO + MI operates in highly automated and prediction-dependent motor skills. During the take-off and early flight phases, athletes have limited time to correct their movements through ongoing feedback and must instead rely on anticipatory control and refined internal models to predict the sensory consequences of action. In elite ski jumpers, these internal action models are likely to become highly automatized through long-term training. AO + MI may therefore be especially relevant in this context because it requires athletes to couple externally observed movement cues with internally generated kinesthetic representations, potentially engaging cortical processes related to sensorimotor prediction, visuomotor transformation, and sport-specific action representation more strongly than either AO or MI alone. Therefore, examining AO, MI, and AO + MI in elite ski jumpers may help clarify how combined motor simulation engages cortical networks related to sport-specific action representation and integrated motor control.

Functional near-infrared spectroscopy (fNIRS) provides a practical method for investigating cortical hemodynamics in ecologically valid settings. Its tolerance to body movement, adequate spatiotemporal resolution, and ease of application make it particularly suitable for studying brain activity in athletes without interfering with task performance [14,15]. Within this framework, the simultaneous coupling of external visual action information and first-person kinesthetic imagery was expected to preferentially recruit frontoparietal regions involved in action representation, visuomotor transformation, and higher-order motor control. In this study, we used fNIRS for the first time to compare cortical activation patterns in professional ski jumpers under three conditions: AO, MI, and concurrent AO + MI. Based on the DASH model and previous neuroimaging findings, we hypothesized that (1) AO + MI would elicit significantly greater activation in frontoparietal regions (including the premotor, prefrontal, and parietal cortices) compared to baseline and that (2) AO + MI would produce stronger hemodynamic responses than either AO or MI alone, reflecting a potential synergistic effect of combined simulation; in addition, (3) athlete level was included as an exploratory between-subject factor to examine whether AO, MI, and AO + MI-related cortical responses varied within this elite athlete sample, without assuming a specific direction of difference between Master-level and First-class athletes.

## 2. Materials and Methods

### 2.1. Participants

Twenty-seven highly trained Chinese ski jumpers (15 males, 12 females; age: 18.56 ± 1.58 years; training experience: 4.1 ± 2.5 years) participated in this study. An a priori power analysis was conducted using G-Power 3.1.9.7 for the repeated-measures design. The analysis indicated that 28 participants would be required to detect a medium effect with 80% power at α = 0.05. The final sample of 27 participants approached the estimated requirement. According to the Chinese national athlete grading system, all participants were classified as either Master-level or First-class athletes, corresponding to Tier 4 (elite/international level) in the participant classification framework for sports science research. All participants were right-handed, had normal or corrected-to-normal vision, and reported no history of color blindness or neuropsychological disorders. Their imagery ability was assessed using the Movement Imagery Questionnaire—Revised (MIQ-R) [16] and met the inclusion criterion (score ≥ 5), with mean scores of 5.70 ± 0.55 for visual imagery and 5.58 ± 0.58 for kinesthetic imagery (both ≥5), indicating adequate imagery ability. Written informed consent was obtained from all participants prior to the experiment. The study was approved by the Ethics Committee of the Capital Institute of Physical Education and Sports and conducted in accordance with the Declaration of Helsinki (Approval No. 2024A104). Participant characteristics by athlete level are presented in Table 1.

### 2.2. Experimental Equipment

**Figure 1:**
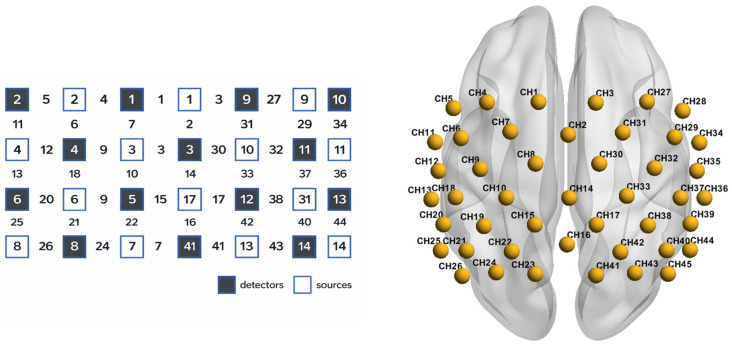
Brain map of the fNIRS probe set, including a table listing the specific brain regions covered according to the international 10 to 20 system.

Hemodynamic responses were measured using a portable functional near-infrared spectroscopy system (NIRSport2, NIRx Medical Technologies, Berlin, Germany) with two wavelengths (760 nm and 850 nm) and a sampling rate of 5.08 Hz. Participants wore a lightweight optical cap containing 14 light sources and 15 detectors, forming 45 measurement channels with a source–detector distance of 3 cm (Figure 1). The optodes were positioned according to the international 10–20 EEG system, and cap placement was adjusted based on anatomical landmarks to improve consistency across participants. Channel locations were estimated with reference to the 10–20 system and converted to MNI coordinates. Anatomical labels were assigned according to the Automated Anatomical Labeling (AAL) atlas. Based on previous neuroimaging studies of AO and MI [5,8], we defined regions of interest (ROIs) in the frontal and parietal cortices, including the prefrontal, premotor, and parietal regions [17,18]. The source–detector pairs, 10–20 positions, MNI coordinates, and anatomical labels for all channels are provided in Supplementary Table S1.

Imagery ability was assessed using the Movement Imagery Questionnaire—Revised (MIQ-R), which consists of eight items rated on a 7-point scale (1 = very difficult to imagine, 7 = very easy to imagine). To monitor participants’ engagement and evaluate the vividness and accuracy of mental representations during the experiment, an Imagery Assessment Scale (adapted from Zhang [19]) was administered immediately after the MI and AO + MI tasks. This scale assessed participants’ first-person kinesthetic imagery use, imagery controllability, and vividness.

The AO task used first-person perspective videos of a master-level athlete performing the ski jumping take-off and flight phases. Each video clip lasted eight seconds and was presented without sound to prevent auditory interference. To reduce visual fatigue, 2–3 different video clips were used.

The MI task required participants to kinesthetically imagine performing the same ski jumping actions. The MI trials were matched to the AO videos in duration and movement content, with each trial lasting 8 s to ensure consistency across conditions. During the MI condition, participants kept their eyes closed and performed first-person kinesthetic motor imagery of the ski jumping approach, take-off, and early flight phases. During the AO + MIcondition, participants watched the video while simultaneously performing first-person kinesthetic imagery of the same movement sequence.

### 2.3. Experimental Protocol

**Figure 2:**
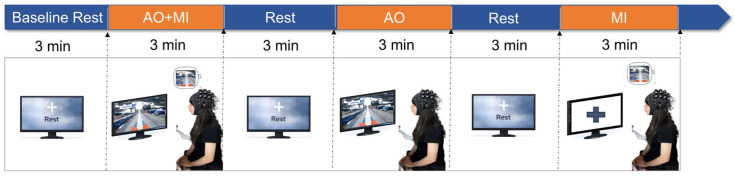
Experimental setup for each condition. Each task block (AO + MI/AO/MI) consisted of 10 trials (8 s task + 10 s ITI).

Prior to the experiment, participants were familiarized with the procedures and completed the MIQ-R. Following the assessment, participants were properly fitted with functional near-infrared spectroscopy (fNIRS) brain imaging equipment, which was maintained throughout the entire session to ensure data continuity. As illustrated in Figure 2, the experimental paradigm was programmed using MATLAB R2018a, (MathWorks, Natick, MA, USA) and the three conditions were presented in a randomized order. Each task followed a block design comprising 10 trials, with each 8 s trial followed by a 10 s inter-trial rest interval. Specifically, the AO + MI task required athletes to watch a ski jumping video while simultaneously performing kinesthetic motor imagery. The AO task involved watching the video only. The MI task required participants to perform first-person kinesthetic motor imagery, focusing on the internal sensations of movement rather than visual representation. Standardized task instructions were provided for all conditions (Supplementary Table S2). Each task session lasted approximately 3 min, with a 3 min rest period between tasks to minimize mental fatigue, resulting in a total experimental duration of 18 min. To monitor imagery engagement, participants completed the Imagery Assessment Scale immediately after all the tasks to evaluate the vividness and accuracy of their mental representations.

### 2.4. fNIRS Data Acquisition and Processing

Raw optical intensity data were first imported into NIRSLAB software, version 2019.4, for preprocessing. Linear detrending was applied to remove low-frequency drift caused by non-neural factors, such as device temperature variations [20]. Motion artifacts were identified and corrected using the preprocessing procedures implemented in NIRSLAB [15]. A band-pass filter (0.01–0.2 Hz) was applied to eliminate high-frequency physiological noise (e.g., cardiac signals) and slow signal drift [21]. Following preprocessing, the optical intensity signals were converted to optical density via logarithmic transformation. Changes in oxygenated hemoglobin concentration (ΔHbO) were calculated using the Modified Beer–Lambert Law (MBLL) [7]. For task-related analysis, the average ΔHbO concentration under each experimental condition was calculated as an indicator of cortical activation. Baseline correction was performed at the trial level, using the mean ΔHbO signal during the 2 s rest period preceding each trial as the baseline. For each trial, the average ΔHbO signal within a time window of 5–8 s after task onset was extracted as the task-related response [22,23]. Changes in oxygenated hemoglobin (ΔHbO) were calculated as the difference between the task-related value and its corresponding baseline [18].

Finally, based on the experimental design, before conducting parametric statistical analyses, the normality of task-related ΔHbO values and paired difference scores was assessed using the Shapiro–Wilk test. One-sample *t*-tests were performed on task-related ΔHbO values to determine whether cortical activation significantly differed from zero under each condition. Paired-sample *t*-tests were conducted to compare ΔHbO differences between experimental conditions. To control for multiple comparisons across the 45 fNIRS channels, *p*-values were corrected using the Benjamini–Hochberg false discovery rate (FDR) procedure, with *q* < 0.05 considered statistically significant. Because minor deviations from normality were observed in a small number of channels, Wilcoxon signed-rank tests were additionally conducted as supplementary sensitivity analyses for the main significant channels. Effect sizes were reported using Cohen’s d for one-sample *t*-tests, Cohen’s dz for paired-sample *t*-tests, and partial eta squared (ηp^2^) for mixed-design ANOVA results. To examine the effect of athlete level, mixed-design ANOVAs were conducted with condition (AO, MI, AO + MI) as a within-subject factor and athlete level (ML vs. FC) as a between-subject factor for selected channels showing significant effects. All statistical analyses were performed using IBM SPSS Statistics, version 25.0 (IBM Corp., Armonk, NY, USA). Brain networks were visualized using BrainNet Viewer, version 1.61 (National Key Laboratory of Cognitive Neuroscience and Learning, Beijing Normal University, Beijing, China) [24].

## 3. Results

### 3.1. Questionnaire Data

Participants’ average visual representation score was 5.06 ± 0.74 and the average kinesthetic representation score was 5.02 ± 0.56, corresponding to moderate to high imagery ease on the 7-point scale. These results indicate that participants maintained adequate imagery ability throughout the experiment.

### 3.2. fNIRS Results

#### 3.2.1. Task-Related Activation Relative to Baseline

**Figure 3:**
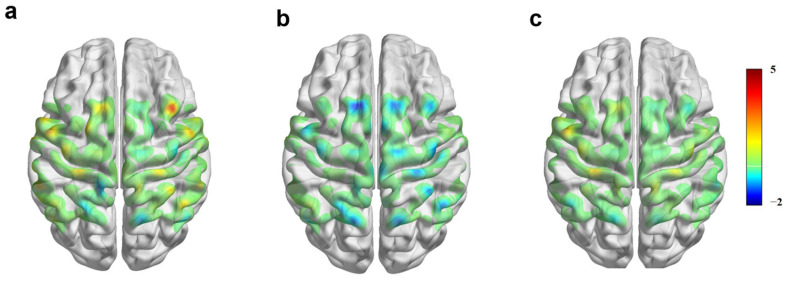
(**a**) Brain activation visualization of the AO + MItask; (**b**) brain activation visualization for MI task; (**c**) brain activation visualization for the AO task.

Figure 3 presents activation patterns based on channel-specific t-values. One-sample *t*-tests against baseline revealed significant activation only in the AO + MI condition (FDR-corrected *q* < 0.05), while neither AO nor MI showed significant activation in any channels after correction. In the AO + MI condition, channels ch5 (t = 4.68, *q* < 0.001, Cohen’s d = 0.90), ch11 (t = 4.41, *q* < 0.001, Cohen’s d = 0.85), and ch27 (t = 4.12, *q* < 0.001, Cohen’s d = 0.79) showed significant positive activation, while no other channels reached significance. These channels correspond to the precentral gyrus (PreCG), postcentral gyrus (PosCG), and middle frontal gyrus (MFG), indicating that activation was primarily localized in these regions. Wilcoxon signed-rank sensitivity analyses further supported the robustness of the significant AO + MI-related activation in these channels. These findings suggest that only the combined AO + MI condition elicited a robust and measurable frontoparietal activation pattern in elite ski jumpers, whereas single-modality conditions did not reach significance after correction.

#### 3.2.2. Between-Task Comparisons

**Figure 4:**
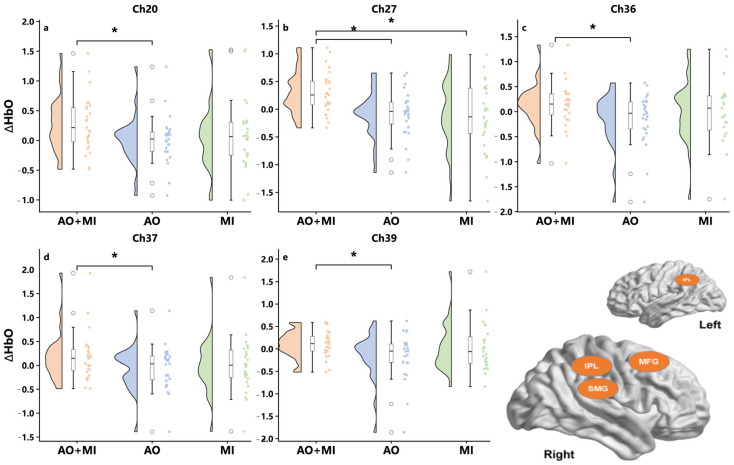
Differential brain activation patterns across three mental simulation tasks. (**a**) ΔHbO responses in Ch20 across AO + MI, AO, and MI conditions; (**b**) ΔHbO responses in Ch27 across AO + MI, AO, and MI conditions; (**c**) ΔHbO responses in Ch36 across AO + MI, AO, and MI conditions; (**d**) ΔHbO responses in Ch37 across AO + MI, AO, and MI conditions; (**e**) ΔHbO responses in Ch39 across AO + MI, AO, and MI conditions. The brain renderings show the anatomical locations of the significant cortical regions, including the supramarginal gyrus, inferior parietal lobule, and middle frontal gyrus. Asterisks indicate significant between-condition differences after FDR correction (*q* < 0.05).

To further investigate differences across task conditions, paired-sample *t*-tests were performed on the ΔHbO values from 45 channels across the three tasks. Channels ch20, ch27, ch36, ch37, and ch39 exhibited significant between-condition differences (FDR-corrected *q* < 0.05; Figure 4). Significant between-condition differences are summarized in Table 2. These channels were primarily located in the supramarginal gyrus (SMG), inferior parietal lobule (IPL), and middle frontal gyrus (MFG), indicating that the differences were mainly concentrated in regions associated with visuomotor integration and higher-order action representation. No channel survived FDR correction in the AO versus MI comparison. Post hoc comparisons revealed that AO + MI elicited significantly greater activation than AO in several channels, whereas its difference from MI was limited to ch27 after FDR correction. This pattern suggests that the combined condition showed a more pronounced advantage over AO than over MI in recruiting frontoparietal regions.

#### 3.2.3. Modulation by Expertise Level

To further investigate whether task-related activation patterns were influenced by athlete level, additional 2 (ML vs. FC) × 3 (AO, AO + MI, MI) mixed-design ANOVAs were conducted on the key channels identified in the main analyses (ch20, ch27, ch36, ch37, and ch39). Across all channels, no significant main effect of level or Condition × Level interaction was observed (all *q* > 0.05, Table 3). These results indicate that the relative pattern of activation across conditions was comparable between the two elite subgroups.

## 4. Discussion

This is the first study to use fNIRS to investigate cortical activation patterns in elite ski jumpers during AO, MI, and combined AO + MI tasks. First, we found that the combined AO + MI task elicited significant cortical activation relative to baseline after FDR correction, whereas AO or MI alone did not produce significant activation compared to the resting baseline. During AO + MI, significant activation was observed in channels corresponding to the MFG, PreCG, and PosCG. Second, between-condition comparisons showed that AO + MI elicited stronger ΔHbO responses than AO alone in several frontoparietal channels, including regions corresponding to the MFG, IPL, and SMG. However, the difference between AO + MI and MI was more limited, with only ch27 surviving FDR correction. No channel survived FDR correction in the AO versus MI comparison. Lastly, additional analyses showed no significant modulation by athlete level, suggesting that the relative advantage of AO + MI was comparable between ML and FC athletes. Overall, these findings suggest that, in elite ski jumpers, concurrent observation and imagery may be particularly effective in recruiting cortical processes relevant to action representation, especially when compared with AO alone. These results provide new insights into the highly specialized brain circuits of elite athletes and may help inform future training-related applications.

Our findings are broadly consistent with the dual action simulation hypothesis (DASH) [12,25], which proposes that when kinesthetic imagery and visual observation focus on the same action, two parallel streams of information converge and potentiate each other, resulting in synergistic neural activation—a “1 + 1 > 2” [12] effect. In this study, this advantage was not diffusely distributed across all measured channels but was concentrated in a limited subset of frontoparietal sites. This pattern of localized activation suggests that the AO + MI interaction may preferentially activate task-relevant brain regions rather than causing a general increase in cortical activity. First, significant activation was observed only under AO + MI conditions, primarily distributed across the MFG, PreCG, and PosCG. Second, the PreCG has been implicated in motor preparation and action representation [26], while the PosCG may contribute to somatosensory processing and the internal simulation of bodily states [27]. The synergistic activation of these two regions under the AO + MI condition supports the DASH model. This is particularly important in the context of ski jumping, as the take-off and initial flight phases rely on the fine integration of postural control, timing of force application, and whole-body coordination [3,28]. Furthermore, the involvement of the MFG further suggests that AO + MIdoes not rely solely on repeated activation of the sensorimotor system but also engages higher-order cognitive control processes necessary for maintaining a stable internal action representation [13,16,29,30].

These between-condition differences were mainly observed in channels corresponding to the MFG, IPL, and SMG, regions that are widely involved in higher-order motor processing [31,32,33,34]. The MFG is commonly associated with executive control, task maintenance, and top-down modulation of motor-related representations [35]. Therefore, the increased recruitment of this region during the AO + MI condition may be attributed to the additional cognitive demands placed on ski jumpers as they process external visual motion cues while maintaining an endogenous kinesthetic representation. Meanwhile, the IPL and SMG have been linked to body schema, multisensory integration, and visuomotor transformation [36,37]. Therefore, the stronger activation observed during AO + MI relative to AO may reflect the additional requirement to integrate external visual motion information with an internally generated kinesthetic representation. It should be noted that the advantage of AO + MI was more evident when compared with AO than when compared with MI. In channels 20, 36, 37, and 39, AO + MI did not differ significantly from MI, suggesting that MI alone may already be an efficient form of motor simulation in elite ski jumpers. Unlike AO, MI requires active first-person motor simulation and may directly engage internal action representations related to planning, sensorimotor prediction, and movement execution [7]. For elite ski jumpers with highly practiced ski jumping representations, MI may therefore access these internal motor models efficiently. In contrast, AO + MI may provide additional benefit primarily by coupling these internal kinesthetic representations with externally observed movement cues. Thus, the advantage of AO + MI may lie less in a general increase in activation and more in the integration of visual and kinesthetic components of sport-specific action representation.

The absence of statistically significant activation during AO and MI after FDR correction may be compatible with the “neural efficiency hypothesis” [38]. According to this hypothesis, highly skilled individuals may show reduced cortical activation during domain-specific tasks, potentially reflecting more efficient neural processing [39]. For highly automated movements, and considering that the take-off and flight phases in ski jumping are highly practiced and temporally structured, a single modality of mental simulation may be sufficient to support motor representation. This may require fewer additional cortical resources and may result in smaller or more spatially restricted ΔHbO changes that do not survive correction for multiple comparisons. However, AO + MI imposes higher demands on parallel processing and integration, increasing the overall cognitive load and making it easier to detect significant hemodynamic changes in the fronto-parietal network. Nevertheless, because the present study did not include novice or intermediate comparison groups and did not measure behavioral performance changes, the findings cannot provide direct evidence for neural efficiency. Future studies should include athletes across a broader range of expertise and combine neural measures with behavioral outcomes to examine this interpretation more directly.

Another notable finding is that athlete level did not significantly influence the observed task effects. In the primary channels, neither the main effect of rank nor the interaction between condition and rank reached statistical significance. This suggests that the relative advantage of AO + MI was comparable between ML and FC athletes. Rather than weakening the main conclusion, this result reinforces the interpretation that the effect of AO + MIis not limited to the highest-ranking group but reflects a mechanism consistently present among top athletes. Since both subgroups consisted of highly trained ski jumpers, their neural representations of the task were likely already relatively homogenized. In this context, the shared high training status may have had a greater influence on cortical responses to the present paradigm than the official athlete rank. Nevertheless, future studies including athletes across a broader skill-level continuum could further examine how AO, MI, and AO + MIeffects vary with expertise.

Several limitations should be noted. First, the sample size was modest due to the limited availability of elite ski jumpers, which may reduce statistical sensitivity and prevent detection of subtle activation in AO or MI conditions. Second, the fNIRS montage covered only frontoparietal regions and did not include short-separation channels, limiting measurement of other cortical areas and preventing explicit removal of superficial physiological signals. Third, behavioral performance was not assessed, and imagery vividness ratings were collected post-task rather than condition-specifically. Training routines and recent injury history were also not systematically recorded. Future studies could include larger samples, broader skill-level comparisons, behavioral outcomes, and condition-specific imagery assessments to further validate and extend these results.

## 5. Conclusions

This study used fNIRS technology to investigate brain activation patterns in elite ski jumpers during AO, MI, and their concurrent combination (AO + MI). The results showed that only AO + MI exhibited significant activation compared to baseline, while AO alone and MI alone showed no significant differences after correction for multiple comparisons. The differences between conditions were primarily observed in channels corresponding to the middle frontal gyrus, inferior parietal lobule, and supramarginal gyrus, suggesting that AO + MI more effectively engaged processes involved in movement representation and visuomotor integration within the parietal lobule. These findings suggest that concurrent AO + MI may more effectively activate cortical processes related to action representation and visuomotor integration in elite ski jumpers compared to single-modality simulation.

## Figures and Tables

**Table 1:** Participant characteristics.

Variable	Master-Level (*n* = 13)	First-Class (*n* = 14)	Total (*n* = 27)
Sex (male/female)	9/4	6/8	15/12
Age (years)	19.00 ± 1.96	18.14 ± 1.03	18.56 ± 1.58
Height (cm)	171.88 ± 6.46	163.93 ± 6.47	167.76 ± 7.43
Weight (kg)	55.18 ± 5.12	47.05 ± 4.45	50.96 ± 6.16
Training years	5.54 ± 2.85	2.79 ± 0.97	4.11 ± 2.52

**Table 2:** Significant between-condition differences after FDR correction.

Comparison	Channel	t	*q* Value	Cohen’s dz
AO + MI vs. AO	ch27	4.019	0.020	0.777
AO + MI vs. AO	ch20	3.674	0.024	0.707
AO + MI vs. AO	ch36	3.516	0.024	0.677
AO + MI vs. AO	ch39	3.301	0.032	0.635
AO + MI vs. AO	ch37	3.027	0.050	0.582
AO + MI vs. MI	ch27	3.667	0.050	0.706

**Table 3:** Results of ΔHbO values for main channels.

Channel	Condition F (*p*, ηp^2^)	Level F (*p*, ηp^2^)	Condition × Level F (*p*, ηp^2^)
ch20	3.248	2.879	0.537
	(0.047, 0.115)	(0.102, 0.103)	(0.588, 0.221)
ch27	8.205	0.155	1.392
	(0.001, 0.247)	(0.697, 0.006)	(0.258, 0.053)
ch36	3.859	0.003	0.472
	(0.028, 0.134)	(0.954, 0.000)	(0.627, 0.019)
ch37	1.849	0.270	1.038
	(0.168, 0.069)	(0.608, 0.011)	(0.362, 0.040)
ch39	3.835	0.000	1.897
	(0.028, 0.133)	(0.989, 0.000)	(0.161, 0.071)

## Data Availability

The data are not publicly available due to restrictions specified in the informed consent form and participant privacy considerations. Participants were informed that the collected data would be used solely for research and data analysis purposes, and no personally identifiable information will be disclosed in any public reports or publications arising from this study. If further information is required, please contact the corresponding author.

## Supplementary Materials

The following supporting information can be downloaded at: https://www.mdpi.com/article/10.3390/brainsci16060629/s1, Table S1: Channel-to-region correspondence based on the Automated Anatomical Labeling (AAL) atlas; Table S2: Task instructions.
