# Supplementary material for: Combined Action Observation and Motor Imagery Elicits Superior Frontoparietal Activation in Elite Ski Jumpers: An fNIRS Study
Source: Brain Sci. 2026 Jun 11;16(6):629. doi: 10.3390/brainsci16060629 (PMC13296683; doi:10.3390/brainsci16060629)
Supplement: Supplementary file 1 [file brainsci-16-00629-s001.zip › brainsci-4342568-supplementary.pdf]

Supplementary Material

Combined action observation and motor imagery elicits superior frontoparietal activation in elite ski jumpers: An fNIRS study

Table S1.Channel-to-region correspondence based on the Automated Anatomical Labeling (AAL) atlas.. 2

Table S2.Task Instructions...6

Table S1. Channel-to-region correspondence based on the Automated Anatomical Labeling (AAL) atlas.

Channel	S—D	Position (10–20 system)	MNI Coordinates X	Y	Z	Anatomical Region
ch15	5—5	CP1h	-15.47	-43.65	78.02	Postcentral_L
ch17	5—12	CP2h	15.23	-43.69	78.21	Postcentral_R
ch10	3—5	CCP1	-29.69	-30.16	72.83	Postcentral_L
ch33	10—12	CCP2	30.32	-29.29	73.22	Postcentral_R
ch19	6—5	CP3h	-40.85	-44.38	65.71	Postcentral_L
ch38	12—12	CP4h	40.84	-44.07	64.81	Postcentral_R
ch1	1—1	FC1h	-13.63	17.58	67.41	Supp_Motor_Area_L
ch2	1—3	FCCz	1.16	1.21	70.65	Supp_Motor_Area_R
ch3	1—9	FC2h	14.89	17.23	66.58	Supp_Motor_Area_R
ch6	2—4	FCC3	-52.26	-0.43	50.63	Precentral_L
ch7	3—1	FCC1	-27.67	2.75	68.31	Frontal_Sup_L
ch8	3—3	C1h	-15.12	-13.23	76.92	Precentral_L
ch9	3—4	C3h	-42.68	-15.96	65.16	Precentral_L
ch11	4—2	FCC5	-65.14	-2.44	23.85	Postcentral_L
ch12	4—4	C5h	-63.49	-16.83	41.21	SupraMarginal_L
ch14	5—3	CCPz	1.70	-30.31	75.97	Paracentral_Lobule_R
ch29	9—11	FCC4	54.13	0.03	49.54	Precentral_R
ch30	10—3	C2h	16.61	-13.24	75.67	Frontal_Sup_R
ch31	10—9	FCC2	28.35	2.32	67.33	Frontal_Sup_R
ch32	10—11	C4h	44.14	-15.40	64.22	Precentral_R
ch34	11—10	FCC6	66.48	-2.69	23.61	Postcentral_R
ch35	11—11	C6h	65.29	-16.79	40.93	Postcentral_R
ch16	5—7	CPPz	0.55	-53.51	71.07	Precuneus_R
ch22	7—5	CPP1	-26.99	-57.04	69.37	Parietal_Sup_L
ch23	7—7	P1h	-15.26	-68.31	65.89	Parietal_Sup_L
ch24	7—8	P3h	-34.72	-67.48	56.60	Parietal_Sup_L
ch41	13—7	P2h	15.04	-68.93	66.10	Parietal_Sup_R
ch42	13—12	CPP2	27.19	-57.32	70.67	Parietal_Sup_R
ch43	13—14	P4h	34.35	-67.76	57.09	Parietal_Sup_R
ch4	2—1	FC3h	-39.29	17.44	56.38	Frontal_Mid_L
ch27	9—9	FC4h	40.44	17.71	56.08	Frontal_Mid_R
ch5	2—2	FC5h	-55.99	14.49	33.28	Precentral_L
ch28	9—10	FC6h	58.20	13.20	33.01	Frontal_Inf_Oper_R
ch26	8—8	P5h	-51.65	-69.22	39.96	Angular_L
ch45	14—14	P6h	50.86	-68.20	39.65	Angular_R
ch13	4—6	CCP5	-67.01	-31.07	28.89	SupraMarginal_L
ch18	6—4	CCP3	-49	-48	60	Parietal_Inf_L
ch20	6—6	CP5h	-61.05	-43.48	44.46	Parietal_Inf_L
ch21	6—8	CPP3	-49.53	-56.59	52.64	Parietal_Inf_L
ch25	8—6	CPP5	-62.31	-56.60	28.63	SupraMarginal_L
ch36	11—13	CCP6	69.06	-30.29	28.56	SupraMarginal_R
ch37	12—11	CCP4	57.13	-30.23	55.23	Parietal_Inf_R
ch39	12—13	CP6h	62.09	-43.19	44.59	SupraMarginal_R
ch40	12—14	CPP4	50.16	-56.48	53.50	Parietal_Inf_R
ch44	14—13	CPP6	61.84	-56.25	28.83	Angular_R

**Table S2. Task Instructions**
Prior to the resting period, participants were instructed as follows:
"Please allow your mind to relax, similar to how muscles recover after exercise, and let your brain naturally rest and recharge."
For the motor imagery (MI) condition, participants were instructed:<br>"You are required to actively imagine yourself performing the ski jumping approach, take-off, and early flight phases from a first-person perspective, focusing on the internal sensations of movement."
For the combined action observation and motor imagery (AO+MI) condition, participants were instructed:<br>"You are required to watch the video while simultaneously imagining yourself performing the ski jumping approach, take-off, and early flight phases from a first-person perspective, focusing on the internal sensations of movement."
For the action observation (AO) condition, participants were instructed:<br>"You are only required to watch the video and should not engage in any form of motor imagery."
